# Prognostic significance of Cbx4 expression and its beneficial effect for transarterial chemoembolization in hepatocellular carcinoma

**DOI:** 10.1038/cddis.2015.57

**Published:** 2015-03-12

**Authors:** H-K Jiao, Y Xu, J Li, W Wang, Z Mei, X-D Long, G-Q Chen

**Affiliations:** 1 Institute of Health Sciences, Shanghai Institutes for Biological Sciences, University of Chinese Academy of Sciences/Chinese Academy of Sciences & Shanghai Jiao Tong University School of Medicine (SJTU-SM), Shanghai 200025, China; 2 Department of Pathophysiology, Key Laboratory of Cell Differentiation and Apoptosis of Chinese Ministry of Education, Shanghai Jiao Tong University School of Medicine (SJTU-SM), Shanghai 200025, China; 3 Department of Liver Surgery, State Key Laboratory of Oncogenes and Related Genes, Shanghai Cancer Institute, Ren-Ji Hospital, Shanghai Jiao Tong University School of Medicine (SJTU-SM), Shanghai 200127, China; 4 Department of Pathology, Youjiang Medical College for Nationalities, Baise 533000, Guang-Xi, China

## Abstract

Our recent investigations showed that polycomb chromobox 4 (Cbx4) promotes angiogenesis and metastasis of hepatocellular carcinoma (HCC) through its sumoylating action on hypoxia-inducible factor-1α protein. Here, we attempt to identify the prognostic significances of Cbx4 by a retrospective analyses in 727 cases of HCC patients with and without postoperative transarterial chemoembolization (TACE) or transarterial embolization (TAE). Binary logistic regression tests indicated that Cbx4 is correlated with histological grading, tumor-node-metastasis stage, microvessel density, distant metastasis and hematogenous metastasis of HCC. By univariate and multivariate analyses, we show that Cbx4 is an independent prognostic factor of HCC, and both TAE and TACE treatments have no effects on the overall survival in HCC patients with low Cbx4 expression. More intriguingly, TACE prolongs, while TAE shortens, the overall survival of HCC patients with high Cbx4 expression, indicating that Cbx4 is a good biomarker on decision-making to perform postoperative TACE in HCC patients. Moreover, Cbx4 overexpression enhances while Cbx4 silencing antagonizes doxorubicin-induced cell death of HCC cell lines. In conclusion, Cbx4 is an independent prognostic factor for HCC patients, and the patients with high Cbx4 expression should receive postoperative TACE treatment to improve their survival.

Hepatocellular carcinoma (HCC) is the fifth most common cancer in men and the seventh in women. Because of its high fatality, it has been regarded as the third most common cause of death from cancers worldwide.^1, 2^ To date, surgery remains the only curative modality for HCC. However, long-term prognosis of HCC patients who underwent curative hepatic resection is still far from settled.^3^ In the past years, there has been an explosion in our understanding of the molecular alternations occurring in HCC, through which a series of molecular biomarkers were identified in terms of both their prognostic significances and potentials as therapeutic targets.^4, 5, 6, 7, 8, 9^ It is probable that malignant angiogenesis is among the strongest signals for aberrant pathway activation in HCC, a hypervascular tumor.^10^ Thus, angiogenic cytokines such as vascular endothelial growth factor (VEGF) may play crucial roles in the pathogenesis of HCC. Based on these discoveries, anti-angiogenesis therapies including drugs targeting VEGF, such as sorafenib, has been approved to be used as the first systemic therapy of HCC, marking a major milestone in the treatment of advanced HCC. However, anti-angiogenic agents have not been as successful as initially imagined in HCC, mainly because tumors that typically responded initially to anti-VEGF therapies quickly become resistant.^10, 11, 12, 13^ However, transarterial embolization (TAE) and transarterial chemoembolization (TACE) have become the major interventional treatments for HCC patients. Current guidelines recommend that curative hepatic resection is indicated only in patients with early-stage HCC and satisfactory liver function. In contrast, TACE is recommended as the standard treatment of intermediate-stage HCC in Barcelona-Clinic Liver Cancer (BCLC) algorithm.^14, 15^ Although it was reported that TACE was more effective in 3- to 5-cm tumors than in smaller ones,^16^ the long-term survival outcomes of patients managed with TACE do not appear fully satisfactory.^13, 17^ A meta-analysis with low risk of section of bias showed that TACE or TAE did not significantly increase survival of intermediated-stage HCC patients.^18^ Hepatic resection is considered as the most effective treatment for HCC patients,^19, 20, 21^ while TAE/TACE is often performed as a postoperative adjuvant therapy to improve the survival of patients.^22, 23, 24^ However, the efficacy and safety of postoperative TAE/TACE is poorly understood. Meanwhile, because HCC includes a heterogeneous population of patients with varying tumor burdens,^3, 25^ it is important to discern what kinds of HCC and genetic profiles can benefits from postoperative TAE or TACE treatment.

Polycomb chromobox homolog (Cbx) protein, which includes different isoforms such as Cbx2, Cbx4, Cbx6, Cbx7 and Cbx8, is a critical component of polycomb repressive complex 1 (PRC1), which acts together with PRC2 to silence gene expressions by specifically modifying nucleosomal histones.^26, 27, 28, 29^ Besides functioning in PRC1-mediated transcription repression, Cbx4 (also known as polycomb 2, Pc2) is also a SUMO (small ubiquitin-related modifier) E3 ligase,^30^ which can enhance the sumoylation of a more limited repertoire of substrates involved in tumorigenesis.^31^ In an early oligonucleotide microarray-based transcription profile analysis including 50 hepatocellular nodular lesions ranging from low-grade dysplastic nodules to primary HCC, Cbx4 was shown to be among the top clusters of genes that were highly correlated with ostensible biological implications.^32^ More recently, Cbx4 expression was reported to be upregulated in cancer tissues and high Cbx4 expression was correlated with *α*-fetoprotein (AFP) level in serum, tumor size, pathologic differentiation, and TNM (tumor, node, metastasis) stages based on the analysis of 246 cases of HCC specimens.^33^ More intriguingly, our group reported that Cbx4 enhances sumoylations of oxygen-sensitive hypoxia-inducible factor 1 alpha (HIF-1*α*), governing its transcriptional activity,^34^ through which Cbx4 increases VEGF production and promotes angiogenesis and metastasis *in vitro* and *in vivo* in HCC.^34, 35, 36^ Accordingly, Cbx4 expression has a significant positive correlation with VEGF expression in a cohort with 727 cases of HCC specimen.^34^ Herein, we continue to investigate the prognostic significance of Cbx4 expression in this cohort of HCC patients with or without postoperative adjuvant TAE and/or TACE treatment.

## Results

### Univariate analyses identify Cbx4 expression as a significant prognostic predictor for survival of HCC patients

Although we and others previously showed that high Cbx4 expression was significantly correlated with poor overall survival (OS) of HCC patients,^33, 34^ it remains to be addressed whether Cbx4 expression is an independent prognostic factor for HCC patients. For this purpose, we performed the univariate analyses to test the relationships between relatively expressed Cbx4 levels by immunohistochemistry (IHC) as described previously^34^ and standard variables to OS or disease-free survival (DFS) in our previously reported cohort of 727 cases of HCC patients. Of note, IHC scores were made for all Cbx4 proteins, which were detectable in both nucleus and cytoplasm in HCC tumor tissues. As shown in Table 1, genders, nationalities, HBsAg, anti-HCV, cirrhosis, drinking and smoking status, tumor number and AFP levels were not associated with survival, while Cbx4 expression (*P*=5.4 × 10^−9^ for OS, *P*=0.001 for DFS), microvessel density (MVD; *P*=5.6 × 10^−5^ for OS, *P*=0.028 for DFS) and histological grade (*P*=2.6 × 10^−6^ for OS, *P*=0.041 for DFS) were among the significant prognostic factors for both OS and DFS in HCC patients. Notably, tumor size and TNM stage were only associated with OS (*P*=0.0002 and *P*=0.001, respectively).

### Cbx4 expression is positively correlated with histological grading and metastasis of HCC

**Figure 1:**
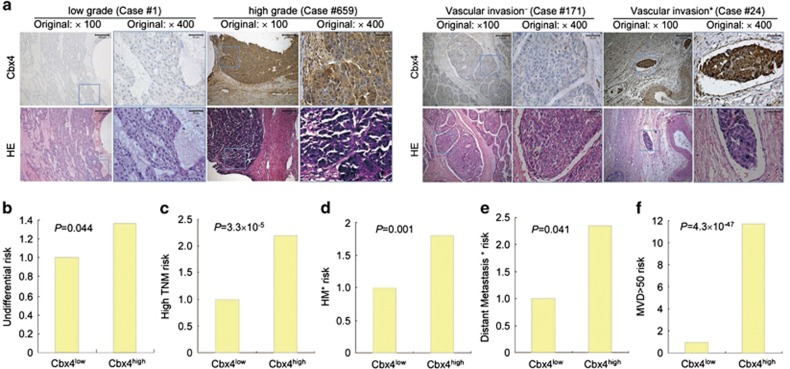
Cbx4 expression is positively correlated with histological grading and metastasis of HCC. (**a**) The representative images for Cbx4 expression and H&E staining in tumors with low and high grade and with or without vascular invasion. (**b**–**f**) The undifferential risk (**b**), high TNM stage (**c**), HM^+^ risk (**d**), distant metastasis risk^+^ (**e**) and high MVD risk (**f**) were compared between tumors with high and low Cbx4 expression in 727 cases of HCC patients. Corresponding risk value odds ratio is calculated using binary logistical regression with likelihood-ratio test for forward method

Next, we assessed the potential relationship between Cbx4 expression and standard variables of HCC patients by *χ*^2^ test. The results showed that Cbx4 expression was positively correlated with histological grading, MVD and TNM stage as well as distant metastasis and hematogenous metastasis (HM), two HCC features obtained from the end of follow-up interviews or the last follow-up interview before death (Table 2). Cancer tissues with high histological grade (Figure 1a) presented high scores of Cbx4 staining, and cancer tissues with high Cbx4 expression presented more vascular invasion (Figure 1a). Moreover, higher Cbx4 expression increased the undifferential risk of HCC with an odds ratio (OR) of 1.365 (95% CI 1.008–1.849, *P*=0.044; Figure 1b), high TNM risk (OR 2.191, 95% CI 1.505–3.190, *P*=3.3 × 10^-5^; Figure 1c), HM risk (OR 1.797, 95% CI 1.256–2.573, *P*=0.001; Figure 1d), and distant metastasis risk (OR 2.349, 95% CI 1.001–5.454, *P*=0.041; Figure 1e) besides MVD reported previously (OR 11.706, 95% CI 8.170−16.772, *P*=4.3 × 10^−47^; Figure 1f).

### Cbx4 expression is an independent prognostic factor for HCC patients

**Figure 2:**
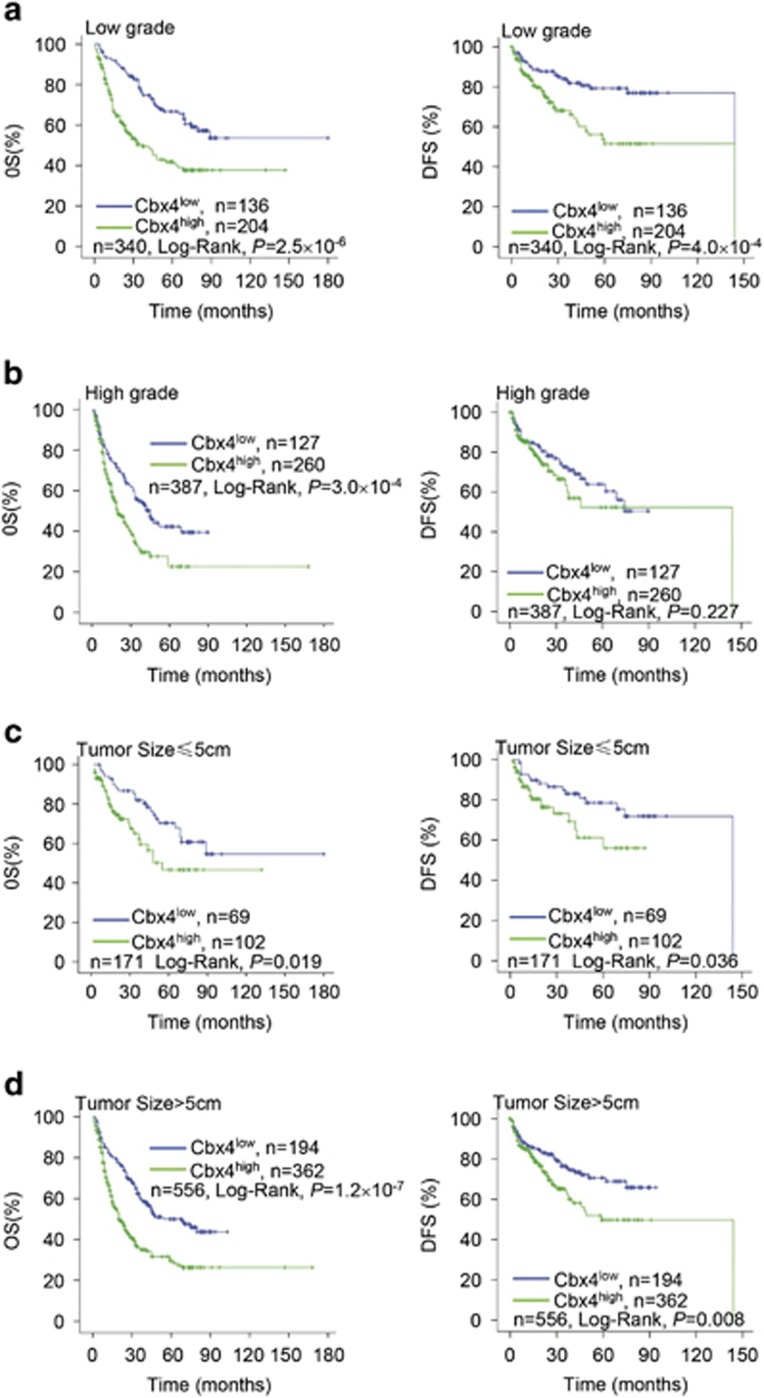
Subgroup analyses for the overall survival (OS) and disease-free survival (DFS) of HCC patients with high and low Cbx4 expression in low grade (**a**) or high grade (**b**) and in tumor size of ≤5 or >5 cm group patients (**c** and **d**)

Because high grade, TNM stage and high MVD were associated with poor clinical outcome, we aimed to determine whether reduced OS and DFS observed in patients with high expression of Cbx4 was an indirect reflection of association between higher Cbx4 expression and these clinicopathological markers or, alternately, whether higher Cbx4 expression might be an independent prognostic factor. To answer this, multivariate analysis based upon Cox proportional hazard regression model was performed. Tumor size (HR 1.875, 95% CI 1.382–2.545, *P*=5.4 × 10^−5^), histological grade (HR 1.588, 95% CI 1.256–2.008, *P*=1.1 × 10^-4^) as well as Cbx4 expression (HR 1.705, 95% CI 1.301–2.233, *P*=1.0 × 10^−4^) were determined as independent prognostic factors for OS. For DFS, histological grade (HR 1.432, 95% CI 1.034–1.983, *P*=0.031) and Cbx4 expression (HR 1.493, 95% CI 1.027–2.170, *P*=0.036) were determined as independent prognostic factors (Table 3). Further stratified analyses based on histological grade and tumor size were performed. In low-grade tumors, high Cbx4 expression had poorer OS (*P*=2.5 × 10^−6^; Figure 2a, left) and DFS (*P*=4.0 × 10^−4^; Figure 2a, right), and Cbx4 expression also presented an inverse correlation with OS outcomes of patients with high-grade tumors (*P*=3.0 × 10^−4^; Figure 2b, left), although no significant statistical difference was found for DFS in high-grade patients with tumors of low and high Cbx4 expression (Figure 2b, right). Also, tumors with high Cbx4 expression had poorer DFS and OS regardless of whether the tumor size was bigger or smaller than 5 cm (Figures 2c and d).

### High Cbx4 expression differentially affects therapeutic effect of TAE and TACE intervention in HCC patients

**Figure 3:**
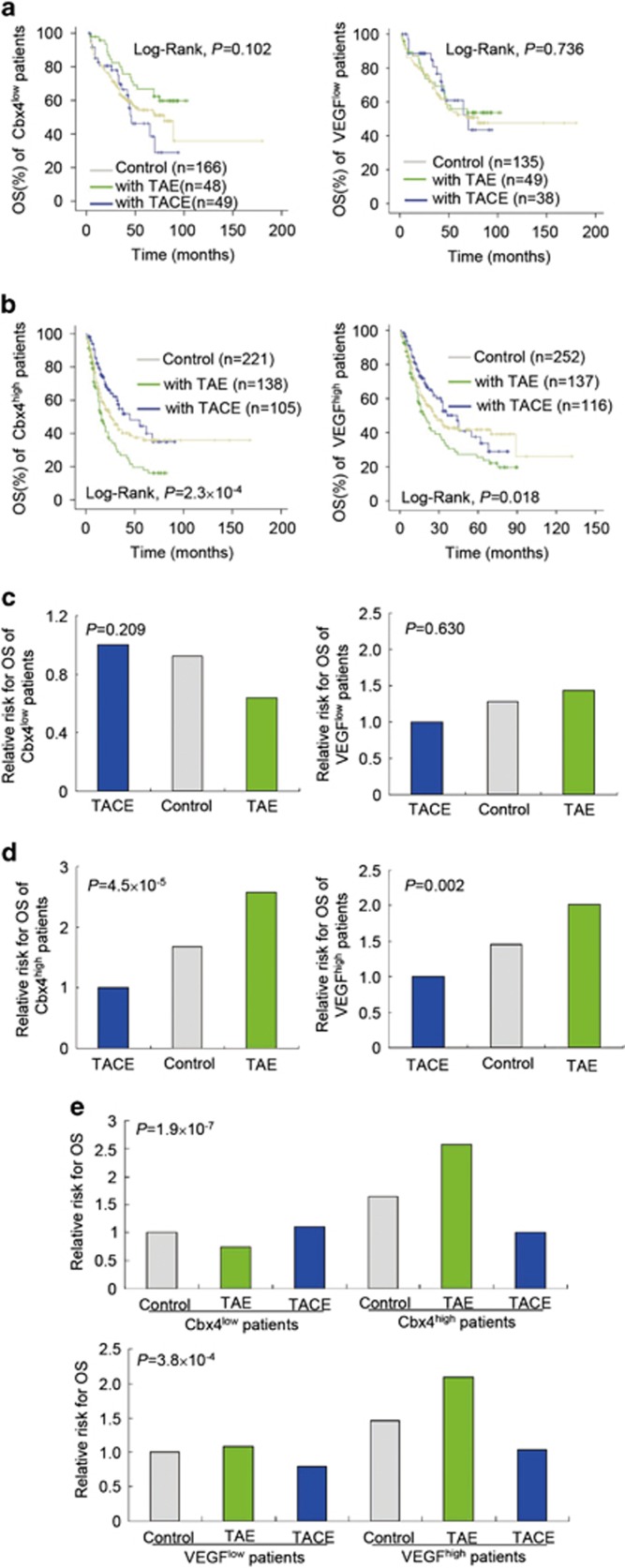
TAE shortens while TACE prolongs the survival of patients with tumors of high Cbx4 or high VEGF expression. (**a** and **b**) Comparison of overall survival among control, TAE and TACE treatment groups of patients with low (**a**) and high Cbx4 or VEGF expression (**b**). The *P*-values for three groups of patients are shown. (**c** and **d**) Comparison of relative risks of death among the indicated three groups of patients with low (**c**) and high Cbx4 or VEGF expression (**d**). The *P*-values for three groups of patients are shown. (**e**) Joint analyses between Cbx4 or VEGF expression and TAE/TACE treatment. The *P*-values for six groups of patients are shown

Based on the fact that highly vascularized tumors are supplied by the hepatic arteries whereas the non-neoplastic liver parenchyma has dual blood supply,^37^ TAE and TACE have gained considerable attentions and been advocated as standard locoregional life-extending treatment for unresectable HCC or as adjuvant therapy after surgery to improve HCC patients' survival.^38^ However, Cbx4 can enhance hypoxia-driven VEGF expression and angiogenesis as a SUMO E3 ligase on HIF-1*α* protein in HCC cells.^34^ Thus, we asked whether Cbx4 and VEGF expression levels impinge on the therapeutic effects of TAE or TACE intervention as adjuvant therapy after surgery in HCC patients. The stratified analysis based on Cbx4 or VEGF expression level showed that TAE and TACE treatment had no effect on the OS of patients with HCC tumors of low Cbx4 or VEGF expression (Figure 3a). More intriguingly, in patients with HCC tumors of high Cbx4 or VEGF expression, TACE prolonged whereas TAE treatment significantly shortened their OS (Figure 3b). In line with this notion, TAE/TACE did not affect the risk of death in patients with HCC tumors of low Cbx4 or VEGF expression (Figure 3c). TACE treatment decreased the risk of death in HCC patients with high Cbx4 expression (TACE *versus* control, HR 0.596, 95% CI 0.410–0.868; *P*=0.007) or high VEGF expression (TACE *versus* control, HR 0.690, 95% CI 0.491–0.969; *P*=0.032), while TAE increased the risk of death in patients with high Cbx4 expression (TAE *versus* control, HR 1.533, 95% CI 1.114–2.110; *P*=0.009) or high VEGF expression (TAE *versus* control, HR 1.396, 95% CI 1.017–1.918; *P*=0.039; Figure 3d). By using control group with low Cbx4/VEGF expression as a reference, further, a joint statistical analysis between Cbx4 or VEGF expression and TAE/TACE treatment on HCC prognosis was performed. The results showed that the cases with HCC tumors of high Cbx4 or VEGF expression would face a decreased risk of death with TACE treatment. However, an increased risk of death was associated with TAE treatment (Figure 3e). These results suggest that Cbx4 as well as VEGF expressions should be able to modify the effects of TAE/TACE treatment to predict the survival of HCC patients.

### Cbx4 overexpression increases doxorubicin-induced death of HCC cells

**Figure 4:**
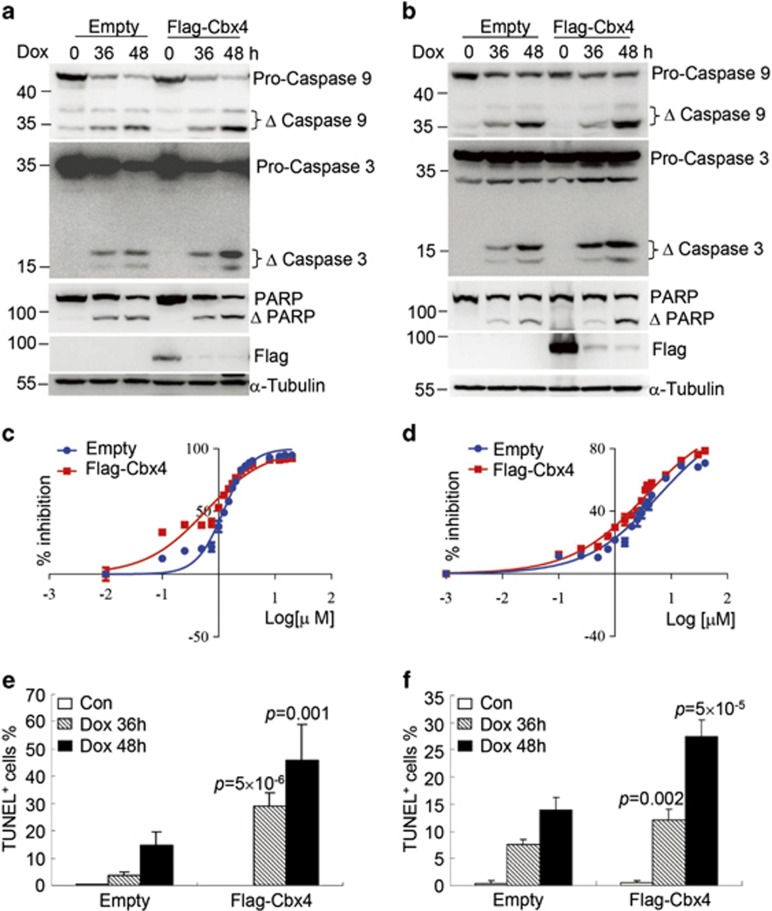
Cbx4 increases the sensitivity of SMMC-7721 and MHCC97L cells to doxorubicin-induced cell death. SMMC-7721 and MHCC97L cells were stably infected with empty or Flag-tagged Cbx4. (**a** and **b**) Doxorubicin was added into SMMC-7721, 2 *μ*M (**a**) and MHCC97L, 3 *μ*M (**b**) cells for 36 and 48 h, followed by immunoblots for the indicated proteins. (**c** and **d**) SMMC-7721 (**c**) and MHCC97L (**d**) with or without ectopic Cbx4 expression were respectively treated with and without different concentrations of doxorubicin for 36 h, and cell growth was assessed by CCK-8 assay and IC50 values of doxorubicin were calculated by GraphPad Prism 6 software. (**e** and **f**) SMMC-7721 (**e**) and MHCC97L (**f**) with or without ectopic Cbx4 expression were treated with 2 *μ*M (**e**) or 3 *μ*M (**f**) of doxorubicin for 36 and 48 h, and percentages of TUNEL-positive cells were calculated

Based on the above-mentioned findings that TACE was beneficial while TAE was harmful to HCC patients with tumors of high Cbx4 expression, we hypothesize that Cbx4 can affect the sensitivity of HCC cells to chemotherapeutic drugs. To address this, two HCC cell lines, SMMC-7721 and MHCC97L, were stably infected with retrovirus carrying Flag alone or Flag-tagged Cbx4 (Figures 4a and b), followed by the treatment with increasing concentrations (from 0.1 to 20 *μ*M for SMMC-7721 (Shanghai, China) and from 0.1 to 40 *μ*M for MHCC97L (Shanghai, China)) of doxorubicin, a commonly used drug in TACE intervention. Thirty-six hours later, CCK-8 assay showed that Cbx4 overexpression significantly increased sensitivity of both HCC cell lines to doxorubicin (Figures 4c and d). The half-maximal inhibitory concentrations (IC50) of doxorubicin were 0.629 *versus* 1.244 *μ*M and 3.414 *versus* 5.969 *μ*M in Flag-tagged Cbx4 and empty vector-infected SMMC-7721 and MHCC97L cells, respectively. Furthermore, Cbx4 overexpression significantly increased doxorubicin-induced cell death in these two cell lines, as assessed by TUNEL assay (Figures 4e and f) and proteolytic activations of caspases 9 and 3 as well as poly(ADP-ribose)polymerase (PARP) cleavage (Figures 4a and b).

### Cbx4 knockdown antagonizes doxorubicin-induced death of HCC cells

**Figure 5:**
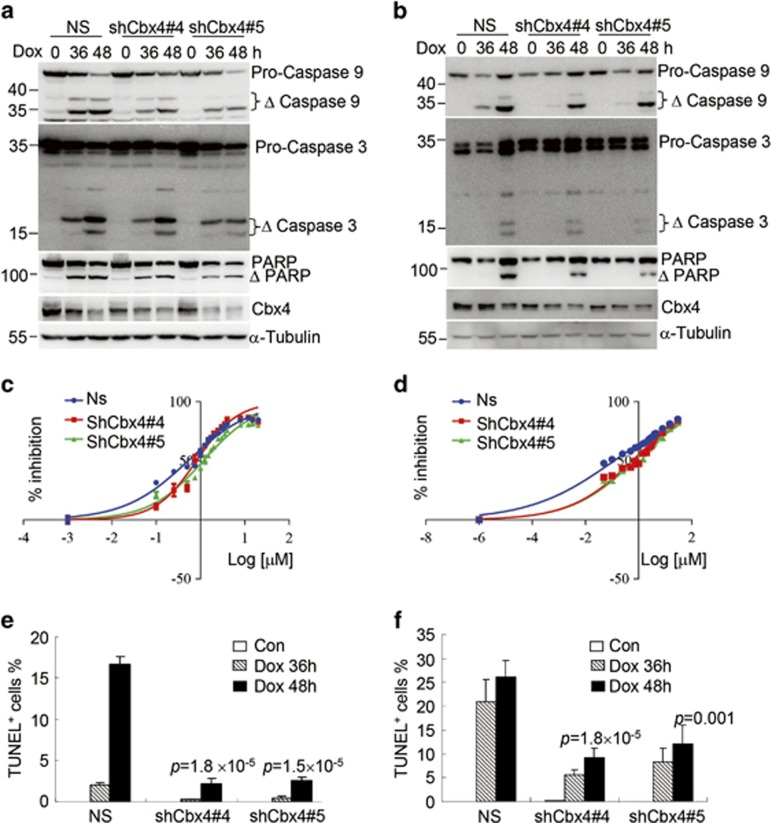
Cbx4 knockdown inhibits the cell death sensitivity of SMMC-7721 and MHCC97L upon doxorubicin treatment. SMMC-7721 and MHCC97L cells were stably infected with two pairs of shRNAs specifically against Cbx4 (shCbx4#4 and shCbx4#5) together with non-specific shRNA as a negative control (NS). (**a** and **b**) Doxorubicin was added into SMMC-7721, 2 *μ*M (**a**) and MHCC97L, 3 *μ*M (**b**) cells for 36 and 48 h, followed by immunoblots for the indicated proteins. (**c** and **d**) SMMC-7721 (**c**) and MHCC97L (**d**) were respectively treated with and without different concentrations of doxorubicin for 36 h, and cell growth was assessed by CCK-8 assay and IC50 values of doxorubicin were calculated by GraphPad Prism 6 software. (**e** and **f**) SMMC-7721 (**e**) and MHCC97L (**f**) with or without Cbx4 knockdown were treated with 2 *μ*M (**e**) or 3 *μ*M (**f**) of doxorubicin for 36 and 48 h, and percentages of TUNEL-positive cells were calculated

Finally, we also knocked down Cbx4 expression in SMMC-7721 and MHCC97L cells using two pairs of shRNAs specifically against Cbx4 (shCbx4#4 and shCbx4#5) together with non-specific shRNA as a negative control (NS). As depicted in Figures 5a and b, the two specific shRNAs could effectively silence Cbx4 expression in these two cell lines. Contrary to what was seen in Cbx4-overexpressed HCC cells, Cbx4 silencing remarkably increased IC50 of doxorubicin in SMMC-7721 cells (Figure 5c), and especially in MHCC97L cells (Figure 5d). Accordingly, Cbx4 knockdown also inhibited doxorubicin-induced cell death, as assessed by TUNEL assay (Figures 5e and f) and cleaved activations of caspases 9 and 3 as well as PARP cleavage (Figures 5a and b). Note that ectopically expressed Cbx4 (Figures 4a and b) and endogenously expressed Cbx4 protein (Figures 5a and b) were decreased during cell death induction.

## Discussion

Here we reported that either TAE or TACE could not improve the survival of HCC patients with low Cbx4 or VEGF expression. However, TAE and TACE presented different effects on the survival of HCC patients with high Cbx4 or VEGF expression. On one hand, TAE treatment shortened the survival of these HCC patients, consistent with the fact that TAE induced ischemic conditions under which Cbx4 enhanced transcriptional activity of HIF-1, promoting the progression of HCC.^34, 39, 40^ On the other hand, TACE treatment could significantly improve the survival of HCC patients with high Cbx4/VEGF expression. All these data suggest that Cbx4 or VEGF can act as an indicator for TACE treatment of HCC patients.

The effects of Cbx4 on tumorigenesis are very complicated. It not only acts as a critical component of PRC1 to contribute to the repression of gene expression by epigenetic modification on chromatin, but also functions as a SUMO E3 ligase to regulate tumorigenesis- or DNA-damage-associated proteins, such as heterogeneous nuclear ribonucleoprotein (hnRNP) K. As reported,^41^ sumoylation of hnRNP K was regulated by the E3 ligase activity of Cbx4, through which DNA damage stimulated hnRNP K sumoylation. The difference of Cbx4 expression on the therapeutic effects of TACE prompted us to investigate why Cbx4 increases the therapeutic benefit of TACE, but not that of TAE, in HCC patients. More intriguingly, we showed that Cbx4 overexpression significantly enhanced, while Cbx4 silencing antagonized, doxorubicin-induced death of HCC cells, supporting the notion that Cbx4 increases the sensitivity of HCC cells to doxorubicin in TACE treatment. The mechanisms underlying Cbx4-increased cell death remain to be further investigated.

In conclusion, our results propose that Cbx4 can act as a biomarker for the prognosis of HCC patients and for predicting the therapeutic effectiveness of TACE on these patients. Furthermore, Cbx4-increased sensitivity of HCC cells to chemotherapeutic drugs together with the Cbx4-increased hypoxia-stimulated angiogenesis^34, 36^ supported the notion that VEGF inhibitor sorafenib in combination with TACE improves the survival of patients,^37, 42^ although more analysis (including prospective study and pathogenesis analysis) deserve further elucidation based on a large sample.

## Materials and Methods

### Patients and specimens

We obtained formalin-fixed and paraffin-embedded tumor specimens of HCC patients, who were histopathologically diagnosed between January 2004 and December 2010 in the Department of Pathology, Affiliated Hospitals of Youjiang Medical College for Nationalities and Guangxi Medical University, as previously described.^34^ All tumors were primary and were untreated before hepatic resection. Retrospective analyses were performed on clinicopathologic data of these 727 HCC patients who underwent curative hepatectomy in the hospital. Of the 727 patients, 186 (25.6%) received postoperative TAE, 154 (21.2%) underwent postoperative TACE, according to Chinese Manage Criteria of HCC,^43^ and the rest (387, 53.2%) received neither after hepatic resection according to the patients' will. The corresponding survival status was confirmed by patients or family contacts. After obtaining written consent, demographic and clinical data were collected in the hospital using a standard interviewer-administered questionnaire and/or medical records. Follow-up interviews ended either by death or on 31 October 2011.

HCC was diagnosed based on evidence of the combination of hepatic angiography, enhanced CT and/or MRI and pathology. Recurrence status was diagnosed by imaging techniques, either intrahepatically or extrahepatically (lymph nodes, distant metastasis). An increased AFP without radiologic evidence was not diagnosed as recurrence until it manifested on imaging. Removed samples of all cases were collected for analyzing the protein expression levels of Cbx4, VEGF and CD31, and H&E staining for pathological analysis was conducted. For histologically differentiated degree of cancer tissues, patients were divided into well and moderately differentiated (low grade), poorly differentiated and undifferentiated (high grade) groups. Classification of TNM stage was assessed according to the sixth edition of the TNM classification system of the American Joint Committee on Cancer/International Union Against Cancer.

To evaluate the role of adjuvant treatment of postoperative TAE/TACE on hepatectomy in patients with low or high Cbx4 expression, all corresponding TAE/TACE treatment information was collected. In this study, TACE consisted of an injection containing a mixture of chemotherapeutic agents and lipiodol followed by embolization with gelatin foam or polyvinyl alcohol until complete stasis was achieved in the tumor-feeding vessels. The chemotherapeutic agents used concurrently included epirubicin, cisplatin and fluorouracil (5-FU). TAE was performed following the same process without chemotherapeutic treatment. According to whether the HCC patients received TAE/TACE treatment, all cases were divided into three groups: (1) TACE, (2) TAE and (3) control groups.

### Immunohistochemistry staining

The protein levels of Cbx4 and VEGF in cells of tumor tissues were analyzed by IHC, respectively with anti-Cbx4 antibody (1 : 25 dilution, SC-19299, Santa Cruz Biotechnology, Dallas, TX, USA) and anti-VEGF polyclonal antibody (1 : 500 dilution, SC-152, Santa Cruz Biotechnology). Angiogenesis was assessed using IHC with anti-CD31 antibody (1 : 50 dilution, Gene Tech, Shanghai, China). All primary antibody stainings were followed by staining with the corresponding horseradish peroxidase (HRP)-conjugated secondary antibodies (KIT-9719 and KIT-9707, Maixin Biotechnology, Fuzhou, China). IHC scores of Cbx4 and VEGF were divided into two classifications: low (immunoreactive scores ≤4) and high (immunoreactive scores >4), according to the value of IRS systems as described in our previous work.^34, 42^ At × 200 magnification, vessel count was made of all distinct brown-staining endothelial cells in the cancerous regions over five fields in each slide. MVD was defined as the average value of three readings. Angiogenesis status was assessed by MVD classification: low (≤50/ × 200 magnification) and high (>50/ × 200 magnification), according to the mean MVD of cancerous-tissue vessels. Images were captured with Nikon Ti-S microscope equipped with a digital camera system (Nikon, Tokyo, Japan).

### Cell culture and transfection

SMMC-7721 were obtained from Cell Resource Center of Shanghai Institute for Biological Sciences, CAS, Shanghai, China. MHCC97L was kindly provided by Dr. Lunxiu Qing at Fudan University (Shanghai, China). These two cell lines were maintained in Dulbecco's Modified Eagle's Medium (DMEM) containing 1% penicillin and streptomycin, supplemented with 10% fetal bovine serum (FBS). Flag-tagged Cbx4, which came from a Cbx4-expressing pCMV plasmid with amino-terminal Flag-tag, generously provided by Dr. Weng JM at East China Normal University (Shanghai, China), was cloned into a pBABE purovector (Cell Biolabs, Inc., San Diego, CA, USA) for stable transfection, as described previously.^34^ Retroviruses were prepared by transient co-transfection with a helper plasmid into HEK293T cells using Lipofectamine 2000 (Invitrogen, Carlsbad, CA, USA) following manufacturer's protocol.

### Western blots

Protein extracts were separated and quantified by 10 and 12.5% SDS-polyacrylamide gel, and transferred to the Immobilon PVDF transfer membranes (Millipore Corporation, Boston, MA, USA). After blocking with 5% nonfat milk in PBS, membranes were immunoblotted with anti-Flag (F1804, Sigma-Aldrich, St. Louis, MO, USA), anti-caspase 3 (#9662, Cell Signaling Technology, Billerica, MA, USA), anti-caspase 9 (#9502, Cell Signaling Technology), anti-PARP-1 (sc-8007, Santa Cruz Biotechnology), anti-Cbx4 (sc-19299, Santa Cruz Biotechnology) antibodies, and then with HRP-conjugated secondary antibody (Cell Signaling Technology) for 2 h at room temperature. Anti-α-tubulin-HRP-conjugated antibody (PM054-7, MBL, Woburn, MA, USA) acted as the internal control. Signals were detected by Immobilon Western chemiluminescent HRP substrate (Millipore Corporation).

### TUNEL assay

Cells were seeded in six-well plates for 24 h and then treated with doxorubicin. After stimulation for 36 and 48 h, the cells in suspension and those attached to the plates were all harvested and resuspended in 600 *μ*l PBS; 100 μl of the cell suspension were subjected to TUNEL staining by using an *in situ* cell death detection kit (Roche, Mannheim, Germany) in combination with 4',6-diamidino-2-phenylindole (DAPI) staining. TUNEL-positive cells were counted in at least 300 cells in randomly chosen fields. The data were expressed as a percentage of TUNEL^+^ cells to total cells.

### Cell counting kit-8 assay

To evaluate sensitivity of HCC cell lines to doxorubicin, the cells were seeded in 96-well plates at a density of 5 × 10^3^ per well for 24 h, followed by treatment with doxorubicin at 18 different concentrations for 36 h. The concentration of doxorubicin ranged from 0.1 to 20 *μ* for SMMC-7721, and from 0.1 to 40 *μ* for MHCC97L. After 34 h of incubation, the cell counting kit reagent were added to the well and incubated for 2 h. Then the absorbance was recorded at 450 nm. The half-maximal inhibitory concentration (IC50) values were calculated by nonlinear regression analysis using the GraphPad Prism software (Version 6.0, GraphPad Software, Inc., San Diego, CA, USA).

### Statistical analysis

All statistical analyses were performed by the Statistical Package for the Social Sciences (SPSS; Version 13.0, SPSS Institute, Chicago, IL, USA). Univariate and multivariate analyses were based on a Cox proportional hazard regression model. The *χ*^2^ test was used to analyze the distribution difference of Cbx4 expression among different clinicopathological features. The corresponding risk value OR and 95% CI were calculated using binary logistical regression with likelihood ratio test for forward method. Kaplan-Meier survival analysis (with log-rank test) was used to evaluate the relationship between Cbx4, VEGF or other clinicopathological factors and HCC prognosis. *P*-values <0.05 were considered statistically significant.

## Figures and Tables

**Table 1:** Univariate analyses identify Cbx4 expression as one of significant prognostic predictors for survival of HCC patients

**Variables**	**Overall survival**		**Disease-free survival**	
	**HR (95% CI)**	***P*-value**	**HR (95% CI)**	***P*-value**
Gender (female *versus* male)	1.050 (0.754–1.462)	0.772	0.954 (0.597–1.525)	0.845
Race (minority *versus* han)	1.138 (0.905–1.431)	0.270	0.879 (0.631–1.224)	0.446
HBsAg (negative *versus* positive)	0.833 (0.596–1.163)	0.283	0.813 (0.510–1.299)	0.387
Anti-HCV (negative *versus* positive)	1.627 (0.228–11.595)	0.627	0.815 (0.114–5.829)	0.839
Hepatocirrhosis (no *versus* yes)	0.959 (0.744–1.235)	0.743	0.792 (0.548–1.145)	0.216
Smoke (no *versus* yes)	1.098 (0.842–1.433)	0.489	1.001 (0.696–1.438)	0.997
Drink (no *versus* yes)	0.879 (0.671–1.150)	0.346	0.985 (0.669–1.450)	0.939
AFP (≥25 *versus* <25 ng/ml)	1.129 (0.907–1.405)	0.277	0.787 (0.581–1.066)	0.122
Tumor number (single *versus* multiple)	1.276 (0.995–1.636)	0.055	1.311 (0.927–1.854)	0.125
Tumor size (≥5 *versus* <5 cm)	2.001 (1.493–2.682)	0.0002	1.315 (0.918–1.885)	0.135
TNM stage (I–II *versus* III *versus* IV)	1.381 (1.137–1.678)	0.001	1.142 (0.863–1.510)	0.353
MVD (>50 *versus* ≤50)	1.612 (1.278–2.033)	5.6 × 10^−5^	1.438 (1.041–1.986)	0.028
Histological grade (high *versus* low)	1.708 (1.366––2.135)	2.6 × 10^−6^	1.379 (1.014–1.875)	0.041
Cbx4 (high *versus* low)	2.009 (1.589–2.540)	5.4 × 10^−9^	1.727 (1.248–2.388)	0.001

Abbreviations: CI, confidence interval; HR, hazards ratio

Univariate analyses were calculated by the Cox proportional hazards regression model

**Table 2:** Cbx4 expression is positively correlated with histological grading and metastasis of HCC

**Variables**	**Cases**	**Cbx4 expression, cases (%)**		***χ*^2^**	***P*****-value**
		**Low**	**High**		
Total	727	263 (100)	464 (100)		
*Gender*				0.450	0.502
Female	88 (12.1)	29 (11.0)	59 (12.7)		
Male	639 (87.9)	234 (89.0)	405 (87.3)		
*Race*				0.407	0.524
Han	478 (65.7)	169 (64.3)	309 (66.6)		
Minority	249 (34.3)	94 (35.7)	155 (33.4)		
*HBsAg*				0.516	0.473
Negative	91 (12.5)	36 (13.7)	55 (11.9)		
Positive	636 (87.5)	227 (86.3)	409 (88.1)		
*Anti-HCV*				3.538	0.060
Negative	725 (99.7)	261 (99.2)	464 (100)		
Positive	2 (0.3)	2 (0.8)	0 (0)		
*Hepatocirrhosis*				0.007	0. 932
No	181 (24.9)	65 (24.7)	116 (25.0)		
Yes	546 (75.1)	198 (75.3)	348 (75.0)		
*Smoke*				0.108	0.743
No	545 (75.0)	199 (75.7)	346 (74.6)		
Yes	182 (25.0)	64 (24.3)	118 (25.4)		
*Drink*				0.058	0.809
No	577 (79.4)	210 (79.8)	367 (79.1)		
Yes	150 (20.6)	53 (20.2)	97 (20.9)		
*Tumor size (cm)*				1.688	0.194
≤5	171 (23.5)	69 (26.2)	102 (22.0)		
>5	556 (76.5)	194 (73.8)	362 (78.0)		
*Tumor number*				1.083	0.298
Single	545 (75.0)	203 (77.2)	342 (73.7)		
Multiple	182 (25.0)	60 (22.8)	122 (26.3)		
*AFP (ng/ml)*				0.297	0.586
<25	311 (42.8)	116 (44.1)	195 (42.0)		
≥25	416 (57.2)	147 (55.9)	269 (58.0)		
*Histological grading*				4.045	0.044
Low	340 (46.8)	136 (51.7)	204 (44.0)		
High	387 (53.2)	127 (48.3)	260 (56.0)		
*Hematogenous metastasis*				10.413	0.001
Absent	157 (21.6)	74 (28.1)	83 (17.9)		
Present	570 (78.4)	189 (71.9)	381 (82.1)		
*Distant metastasis*				4.167	0.041
No	692 (95.2)	256 (97.3)	436 (94.0)		
Yes	35 (4.8)	7 (2.7)	28 (6.0)		
*MVD*				207.745	4.3 × 10^−47^
≤50	261 (36.0)	184 (70.0)	77 (16.6)		
>50	465 (64.0)	79 (30.0)	387 (83.4)		
*TNM stage*				17.895	1.3 × 10^−4^
I–II	138 (19.0)	71 (27.0)	67 (14.4)		
III	518 (71.3)	172 (65.4)	346 (74.6)		
IV	71 (9.8)	20 (7.6)	51 (11.0)		

**Table 3:** Independent prognostic factors of OS and DFS for HCC patients by multivariate analyses

**Variables**	**Overall survival**		**Disease-free survival**	
	**HR (95% CI)**	***P*-value**	**HR (95% CI)**	***P*-value**
Gender (female *versus* male)	1.003 (0.711–1.415)	0.986	1.039 (0.639–1.688)	0.878
Race (minority *versus* han)	1.054 (0.835–1.330)	0.657	0.864 (0.617–1.210)	0.395
HBsAg (negative *versus* positive)	0.884 (0.629–1.242)	0.477	0.878 (0.546–1.412)	0.592
Anti-HCV (negative *versus* positive)	1.047 (0.142–7.705)	0.964	0.604 (0.080–4.557)	0.625
Hepatocirrhosis (no *versus* yes)	0.957 (0.740–1.238)	0.738	0.798 (0.549–1.161)	0.238
Smoking (no *versus* yes)	1.354 (0.927–1.979)	0.117	0.981 (0.590–1.631)	0.941
Drinking (no *versus* yes)	0.702 (0.478–1.031)	0.071	1.031 (0.600–1.771)	0.912
AFP (≥25 *versus* <25 ng/ml)	1.017 (0.810–1.276)	0.885	0.730 (0.532–1.000)	0.050
MVD (>50 *versus* ≤50)	1.287 (0.985–1.682)	0.064	1.215 (0.838–1.762)	0.305
Tumor number (single *versus* multiple)	0.948 (0.724–1.240)	0.694	1.148 (0.783–1.681)	0.480
TNM stage (I–II *versus* III *versus* IV)	1.138 (0.911–1.420)	0.255	1.007 (0.740–1.371)	0.962
Tumor size (≥5 *versus* <5 cm)	1.875 (1.382–2.545)	5.4 × 10^−5^	1.244 (0.848–1.825)	0.263
Histological grading (high *versus* low)	1.588 (1.256–2.008)	1.1 × 10^−4^	1.432 (1.034–1.983)	0.031
Cbx4 (high *versus* low)	1.705 (1.301–2.233)	1.0 × 10^−4^	1.493 (1.027–2.170)	0.036

Abbreviations: CI, confidence interval; HR, hazards ratio

Multivariate analysis was performed using the Cox multivariate proportional hazards regression model with stepwise manner (forward, likelihood ratio)

## Abbreviations

AFP: α-fetoprotein
CI: confidence interval
DFS: disease-free survival
DM: distant metastasis
HCC: hepatocellular carcinoma
HM: hematogenous metastasis
HR: hazards ratio
MVD: microvessel density
OR: odds ratio
OS: overall survival
TACE: transarterial chemoembolization
TAE: transarterial embolization
TNM: tumor-node-metastasis
VEGF: vascular endothelial growth factor
